# Putting one health to the test: Operational challenges and critical reflections from the global South

**DOI:** 10.1016/j.onehlt.2024.100963

**Published:** 2024-12-29

**Authors:** Mridula Mary Paul, Sunita Pradhan, Aarti Chettri, Sarala Khaling, Abi T. Vanak

**Affiliations:** a Department of Geography & Environmental Sciences, Northumbria University, Ellison Building, Newcastle-upon-Tyne NE1 8ST, UK; b Department of Zoology, Sikkim Government College, Namchi, Sikkim 737126, India; c Ashoka Trust for Research in Ecology and the Environment, Regional Office-Northeast India, Gangtok, Sikkim 737101, India; d Centre for Policy Design, Ashoka Trust for Research in Ecology and the Environment, Bengaluru, India & School of Life Sciences, University of KwaZulu-Natal, Durban, South Africa

**Keywords:** One health, Zoonoses, Health policy, Structural violence of policies

## Abstract

One Health as a policy framework to tackle zoonoses has gained wide-ranging validation with multiple international organizations throwing their collective might behind it. Such endorsement has convinced several governments to adopt One Health as a national strategy to address zoonoses. Although some argue that One Health is so many things that there are in fact multiple ‘One Healths’, others find that most international policy documents that use the One Health framing contain certain key recommendations, with intersectoral coordination and disease surveillance prominent among them. In this paper we examine whether and how One Health travels in a sub-national setting in a developing country context such as that of India, with particular focus on intersectoral coordination. We draw on documentary analysis, semi-structured interviews, and workshops with government officials across key sectoral agencies at the district level, to understand prevalent institutional mechanisms in place to address zoonoses in such a setting. We locate our study in the district of Gyalshing in the state of Sikkim in India, which is a potential zoonoses ‘hotspot’ given its location within the biodiverse Indian Himalayan Region, with numerous avenues for human-animal interactions, and burgeoning human population linked to its tourism-run economy. We outline successful cases where certain zoonotic diseases could be tackled, while also highlighting structural constraints that need to be borne in mind while planning or advocating One Health as a blanket policy prescription. In doing so, we draw attention to the political dimensions of global health policies, and question whether One Health can be uncritically deployed in developing country contexts.

## 1 Introduction

One Health came into prominence as a policy prescription in the wake of the avian influenza pandemic scare in the early 2000s [1], and was subsequently endorsed by a number of global institutions [2,3]. Although wildlife reservoirs of zoonoses were of concern in these earlier policies, COVID-19 provided a policy window like no other. It was the zoonotic pandemic that avian influenza had long threatened to be. In the light of COVID-19's suspected wildlife origin, global policies to tackle zoonoses now clearly relied on a One Health framing [3]. More recently, with the endorsement of the United Nations Environment Programme, environmental health has been more firmly incorporated into a global One Health policy framing [4,5].

Although there is no singular One Health framework, but instead a wide array of policies that all draw on varied versions of One Health [6], one of the key recommendations across global One Health policies is the creation of inter-sectoral One Health coordination mechanisms at the national and sub-national levels [1]. We critically analyze this aspect of One Health, focusing on a sub-national administrative unit in a developing country context. Through an examination of government reports, key informant interviews, and stakeholder workshops, we examine the prevalent institutional structures in place to address zoonoses in a part of India that falls within the Himalayan biodiversity hotspot, which is considered a ‘hotspot’ for the risk of emerging diseases [7]. By exploring the institutional capacity for operationalizing One Health at a sub-national level, we reflect on whether global zoonoses prescriptions are cognizant of the constraints these systems function under in a developing country context, and thereby reflect on uncritically translocating the One Health framework to the global south.

## 2 Locating One Health

In their analyses of the political economy of One Health, Galaz and others [1] outline the power relations contained within the networks and knowledge production systems underlying One Health, clearly placing institutions and actors headquartered in the global North at its heart. Narratives around the control and management of infectious diseases have a long history of emerging from and being dominated by the global North, whether that was in the pursuit of empire-building [8,9] or modern-day global trade [10] and geopolitical dominance [11]. The use of the language of ‘biosecurity’ applied to the global responses to zoonoses draws on this tradition [12], further reinforced in a globalized world with fluid borders by the idea that the “fight must be taken ‘over there’ before it ‘reaches here’” [10].

Inter-governmental One Health guidelines that suggest building laboratory networks in ‘hotspots’ that can feed real-time information on outbreaks to global databases [5] or train communities to reorder their interactions with their livestock to meet biosecurity regulations [10], clearly support strategies of containment, rather than redressal. Hinchliffe et al. [13] argue that One Health adopts a ‘contamination’ approach to tackling zoonoses i.e., a focus on breaking the chain of transmission, rather than a ‘configuration’ approach which sees disease as a relational occurrence produced by a range of social, political, and economic elements interacting outside of just the host–pathogen-environment dynamic.

In doing so it operates as a ‘techno-science solution’ [14] that shifts attention away from structural factors linked to zoonotic diseases such as poor public health facilities [15,16], or environmental degradation posed by dams, deforestation, mining, or industrial food production systems [17,18]. Often the very same global institutions that now promote One Health – the World Bank for instance [2], have actively funded ecological disruptions that have been linked to the emergence of zoonoses [19]. In the next sections, we draw on empirical findings to explore the operational realities of addressing zoonoses in a district in India, thereby examining the applicability of the One Health prescription of intersectoral coordination in such a setting.

## 3 Sub-national One Health competencies

Eastern Himalaya in India (EHI) is part of the Himalaya biodiversity hotspot comprising a largely mountainous territory with elevation ranging from 50 m in the Brahmaputra valley to 7000 m above mean sea level in the Himalayan borderlands [20]. EHI is the transition zone of India with the Indo-Myanmar, Indo-Malayan and Indo-Chinese biogeographical regions [21]. With climatic conditions ranging from sub-tropical to cold temperate, this landscape supports a rich diversity of wildlife, including mammals, and provides habitat for a range of vectors and pathogens that need to be better understood, given that these are ideal conditions for the emergence and re-emergence of zoonotic diseases [7].

Located with EHI in the north-east region of India, the state of Sikkim has a forest cover 47.31 % of its total geographical area [22]. 147 species of mammals [23] are reported from the state, and a network of eight protected areas, covering 31 % of state's geographical area, harbour most of these species. However, as many as 39 % of these mammal species were also recorded from outside protected areas [24]. These socio-ecological landscapes are largely rural and agrarian, partly fringing the forests, and support varied agro-ecosystems and associated livestock [24]. Livestock population in the state has also seen growth, reflected in an increase of 73 % in cattle, 267 % in buffalo and 275 % in backyard poultry between 2007 and 2012 [25,26]. 60 % of households that rear livestock are located in rural areas [25,26]. Tourism is another mainstay of the economy, with arrivals between 2010 and 2017 almost surpassing the state's population [27].

**Fig. 1:**
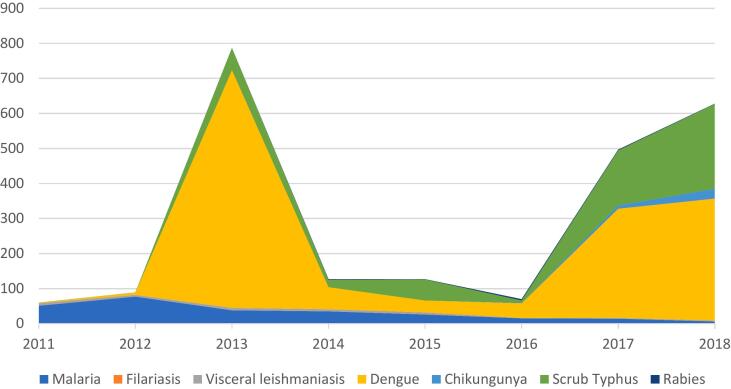
Zoonotic disease prevalence in Sikkim (2013–2018). Source: [1]; [2] 1. DoH, *Annual Report (2020−21) National Vector Borne Disease Control Programme*. 2021, Health and Family Welfare Department, Government of Sikkim: Gangtok. 2. DoAH, *Year-wise Incidence of Rabies and Brucellosis*. 2024, Department of Animal Husbandry and Veterinary Services, Government of Sikkim: Gangtok.

Sikkim records as many as nine zoonotic diseases of concern, which are listed in Table 1. Japanese encephalitis and Nipah virus infection, while being a concern in neighboring states, have not been reported from the state, while only one case of filariasis is recorded so far [28]. Avian influenza, was reported from Namchi District in 2009 [29]. Chakrabarti and others [30] suspect a possible endemicity of avian influenza in the eastern and north-eastern parts of India, demanding active surveillance specifically in view of the critical mutations that have been observed in Influenza A H5N1 viruses. Fig. 1 shows zoonotic disease prevalence in Sikkim from 2011 to 2018. Of particular significance is scrub typhus, which saw an outbreak in the state in 2011 and is on the rise.

**Table 1:** Zoonotic diseases in Sikkim based on literature review.

	Diseases	References
1	Avian flu	[1,2]
2	Brucellosis	[3]
3	Chikungunya	[4]
4	Dengue	[5]
5	Filariasis	[4]
6	Malaria	[4,6]
7	Rabies	[7,8]
8	Scrub typhus	[[9], [10], [11]]
9	Visceral leishmaniasis	[12,13]

1. Chakrabarti, A.K., et al., Characterization of the influenza A H5N1 viruses of the 2008-09 outbreaks in India reveals a third introduction and possible endemicity. PloS one, 2009. **4**(11): p. e7846.

2. DoAHDF, Confirmation of Avian Influenza in South Sikkim District of Sikkim. 2009, Department of Animal Husbandry Dairying & Fisheries, Ministry of Agriculture, Government of India: New Delhi.

3. Shakuntala, I., et al., Incidence of brucellosis in livestock in North-Eastern India. International Journal of Infectious Diseases, 2016. **45**: p. 474.

4. DoH, Annual Report 2018-2019. 2019, Department of Health Care, Human Services, and Family Welfare, Government of Sikkim: Gangtok, Sikkim. p. 202.

5. Bhutia, K.D. and S.C. Lamtha, First outbreak of dengue fever in East Sikkim in Northeastern part of India. Journal of Family Medicine and Primary Care, 2019. **8**(3): p. 1007.

6. Sarma, D.K., et al., Malaria in North-East India: importance and implications in the era of elimination. Microorganisms, 2019. **7**(12): p. 673.

7. Byrnes, H., A. Britton, and T. Bhutia, Eliminating dog-mediated rabies in Sikkim, India: a 10-year pathway to success for the SARAH program. Frontiers in veterinary science, 2017: p. 28.

8. Auplish, A., et al., Estimating the intra-cluster correlation coefficient for evaluating an educational intervention program to improve rabies awareness and dog bite prevention among children in Sikkim, India: A pilot study. Acta tropica, 2017. **169**: p. 62-68.

9. Mohanty, A., et al., Scrub typhus–A case series from the state of Sikkim, India. International Journal of Critical Illness and Injury Science, 2019. **9**(4): p. 194.

10. Gurung, S., J. Pradhan, and P. Bhutia, Outbreak of scrub typhus in the North East Himalayan region-Sikkim: an emerging threat. Indian journal of medical microbiology, 2013. **31**(1): p. 72-74.

11. Gupta, N., et al., Pediatric scrub typhus in South Sikkim. Indian Pediatrics, 2012. **49**(4): p. 322-4.

12. Adhikari, L., et al., Sporadic case of visceral leishmaniasis in Sikkim, India. Journal of Global Infectious Diseases, 2010. **2**(2): p. 196.

13. Maheshwari, G. and A. Chhonkar, Leishmania vector in prospective. Everyman's Science, 2018: p. 39.

**Fig. 2:**
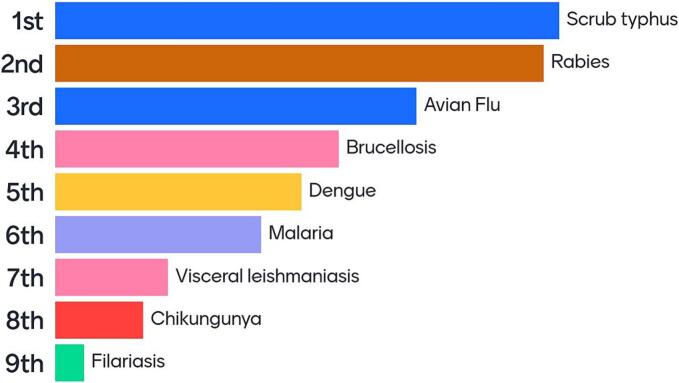
Zoonotic disease prioritization for Gyalshing.

Gyalshing is one of the six administrative districts of the state of Sikkim, with an eponymous district headquarters that is 120 km. away from the state capital at Gangtok – a distance that is infinitely compounded by the mountainous terrain and poor road conditions, plagued by frequent landslides and blockages. Fig. 2 shows zoonotic disease prioritization specific to Gyalshing, with broad overlaps with Fig. 1.

Gyalshing's institutional mechanisms for addressing zoonoses are spread over several government departments. The Department of Health and Family Welfare (DoH) supports 41 primary health sub-centers (PHSCs), 7 primary health centers (PHCs), and one district hospital at Gyalshing. Gyalshing does not have a community health center unlike some other districts of the state [31]. The Department of Animal Husbandry and Veterinary Services (DoAH) has 1 district hospital, 15 veterinary dispensaries, 18 stockman centers, and 5 livestock farms [32]. The Department of Forest and Environment (DoFE) comprises the Directorates for Wildlife Circle, Wildlife Research, and Zoological Parks, under which there is a wildlife veterinary hospital and rescue centre [33].

Apart from these key sectoral agencies, we identified four other departments that addressed zoonoses, directly or in conjunction with these. These include the Department of Agriculture (DoA), Department of Land Revenue and Disaster Management (DoLRDM), and the Departments of Rural and Urban Development (DoRD and DoUD). Further, given the strategic border location of the state of Sikkim, the Armed Forces was found to engage with zoonoses through its Biological Disasters Unit.

## 4 Methodology

Considering the unavailability of literature categorizing zoonoses specific to Gyalshing, we drew on literature from across the state of Sikkim to identify the prevalent zoonotic diseases. Data from DoH for 6 diseases from 2011 to 2018, along with information on scrub typhus from 2014 to 2018 [34], and rabies from 2005 to 2016 [35] was used. This was presented to key officials from DoH, DoAH, and DoFE in Gyalshing and Gangtok through semi-structured interviews (see **Appendix I**) conducted between 01-04-2021 and 09-04-2021, with some clarificatory follow-up phone conversations in August 2022. Adopting a prioritization model [36] in consultation with key informants, 6 criteria were identified to prioritize diseases:

• Intervention availability
• Severity of disease
• Economic burden
• Response capacity
• Transmissibility
• Research and information

**Fig. 3:**
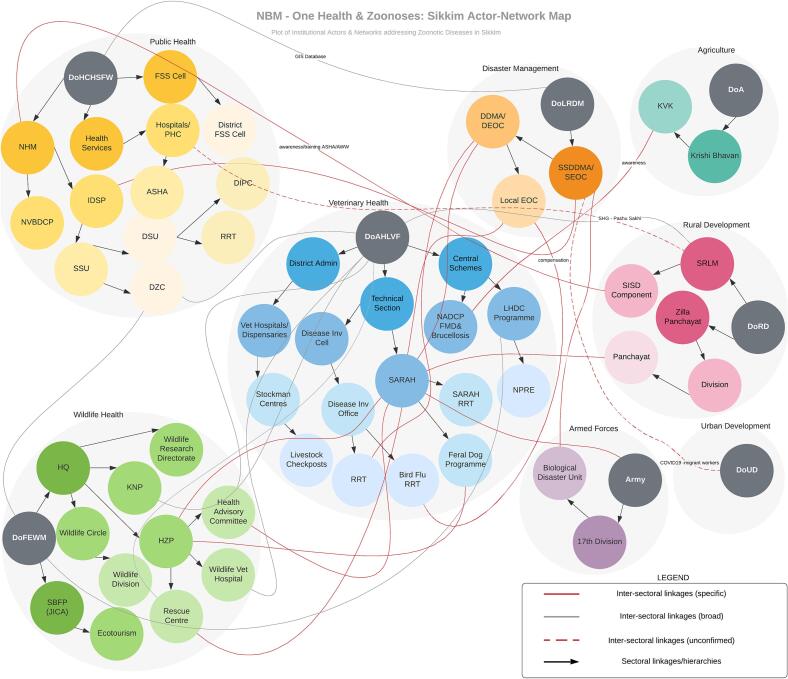
Gyalshing: Institutional networks of One Health & zoonoses. Acronyms: ASHA: Accredited Social Health Activist [community health worker]. AWW: *Anganwadi* Worker [female health and nutrition community worker]. DDMA: District Disaster Management Authority. DEOC: District Emergency Operational Centre. DIPC: District Influenza Epidemic Preparedness Committee. DoA: Department of Agriculture. DoAHLVF: Department of Animal Husbandry Livestock Fisheries & Veterinary Services. DoFEWM: Department of Forest Environment & Wildlife Management. DoHCHSFW: Department of Health Care Human Services & Family Welfare. DoLRDM: Department of Land Revenue and Disaster Management. DoRD: Department of Rural Development. DoUD: Department of Urban Development. DSU: District Surveillance Unit. DZC: District Zoonotic Committee. EOC: Emergency Operational Centre. FSS: Food Safety & Standards. HQ: Headquarter Division. HZP: Himalayan Zoological Park. IDSP: Integrated Disease Surveillance Programme. KNP: Khangchendzonga National Park. KVK: *Krishi Vigyan Kendra* [farm science centre]. LHDC: Livestock Health & Disease Control Programme. NADCP FMD: National Animal Disease Control Programme – Foot & Mouth Disease. NHM: National Health Mission. NPRE: National Programme on Rinderpest Eradication. NVBDCP: National Vector Borne Disease Control Programme. PHC: Primary Health Centre/s. RRT: Rapid Response Team. SARAH: Sikkim Anti-rabies and Animal Health Programme. SBFP (JICA): Sikkim Biodiversity Conservation & Forest Management Project (Japan International Cooperation Agency). SEOC: State Emergency Operational Centre. SHG: Self-help Group. SISD: Social Inclusion & Social Development. SRLM: State Rural Livelihood Mission. SSDMA: Sikkim State Disaster Management Authority. SSU: State Surveillance Unit.

Each of these criteria have questions with assigned weights, which was used to rank the diseases (detailed in **Appendix II**). Key informant interviews were also used to corroborate a detailed institutional map, developed based on government reports, specifying multi- and intra-department structures, programmes, and hierarchies. Inter-departmental interactions and connections were plotted for Gyalshing on a network map using Lucidchart (Fig. 3). These were further corroborated through two stakeholder workshops held in April and August 2021 involving officials from the District Administration, DoH, DoAH, DoFE, DoA, in addition to faculty from Sikkim University and representatives of NGOs working in the area.

## 5 Results

### 5.1 Intersectoral linkages

The institutional network map (Fig. 3) reveals that DoAH is the key government department in tackling zoonoses in the state in terms of the programmes instituted, although many of these are restricted to livestock. It does, however, through the Sikkim Anti-Rabies and Animal Health Programme (SARAH) have a programme targeting feral and wild animals in specific landscapes. In fact, many of the functional inter-sectoral linkages addressing zoonoses emanate from SARAH, which has linkages with DoH, DoLRDM, DoRD, DoA, DoFE, as well as with the Armed Forces. DoAH also interacts with DoA in implementing national livestock health schemes.

The Integrated Disease Surveillance Programme (IDSP) is a national mechanism under the Ministry of Health & Family Welfare for reporting infectious diseases (IDSP 2020). At the state, it is located within DoH, and through its District Zoonotic Committee that has representatives from DoAH, DoEF, and DoLRDM, DoH has institutional linkages with these agencies. Apart from IDSP, DoH does not have additional mechanisms that specifically target zoonoses. However, it does appear to deploy existing community health staff to tackle infectious disease more broadly, through trainings for Accredited Social Health Activists (ASHA) workers and *Anganwadi* Workers (female health and nutrition community workers), in conjunction with DoRD's State Rural Livelihood Mission [37]. The limited involvement of DoFE with programmes to tackle zoonoses reveals a critical lacuna from a One Health perspective.

### 5.2 Surveillance

Occurrences of zoonotic diseases are reported through the IDSP mechanism in Gyalshing through the office of the District Surveillance Officer. Nevertheless, since some of the sub-district units do not have internet connectivity [38], it is unclear how comprehensive the data capture is. DoAH implements the National Animal Disease Control Programme for Foot and Mouth Disease and Brucellosis [39], under the aegis of which it undertakes regular vaccinations that are reported to the Information Network for Animal Productivity and Health platform. However, the limited diagnostic facilities in remoter villages and symptomatic overlaps with other febrile illnesses (MMD, personal communication, April 16, 2021) suggest that cases of brucellosis might be underreported.

The National Animal Disease Reporting System, a national livestock disease reporting platform, is operational in the district although we were unable to ascertain details of processes and coverage. There is no mechanism in place for wildlife health surveillance at the district or sub-district level. There is however a small enclosure managed by the Forest Range Office with the assistance of livestock veterinarians since there are no trained wildlife veterinarians, or facilities for diagnosis or treatment available within the district (TBS, personal communication, August 2, 2022). SARAH's feral dog and wildlife conservation programme undertakes surveillance of sylvatic rabies in cold desert landscapes and national parks.

### 5.3 Response

SARAH has two Rapid Response Teams (RRTs), one of which is specific to the feral dog programme. SARAH also taps into the networks of the District Disaster Management Authority headed by the District Collector, with the support of local governance bodies at the village level. DoAH has two additional RRTs, of which one is specific to avian influenza. The Bird Flu RRT has had success in preventing an outbreak of avian influenza despite one in the neighboring state of West Bengal in 2015. IDSP's District Surveillance Unit has an RRT, as well as a District Influenza Epidemic Preparedness Committee, although the outcomes of their deployment are unclear. The absence of RRTs located within DoFE was keenly felt during a major dermatitis outbreak in gorals and dogs between 2015 and 2016, as reported by participants during the workshops conducted during the study.

### 5.4 Constraints to operationalizing One Health

Similar to observations made at the national level [40], while there is considerable work undertaken to address zoonoses within the state of Sikkim, these are largely disease specific, with each sector focusing on those that fall within their mandate. A fundamental issue in addressing zoonoses at a sub-national level in a state like Sikkim, is the severe limitations posed by the absence of basic infrastructure and institutional capacity. This is particularly evident in the complete absence of wildlife veterinarians and facilities for wildlife disease surveillance. The forest and wildlife department does not possess a response mechanism in the event of an epizootic outbreak. The less obvious sectoral capacity constraint is that of the public health institutions. Even the much-celebrated IDSP remains largely a reporting mechanism, and does not appear to have programme-level inter-sectoral linkages in Gyalshing.

## 6 Discussion

**Fig. 4:**
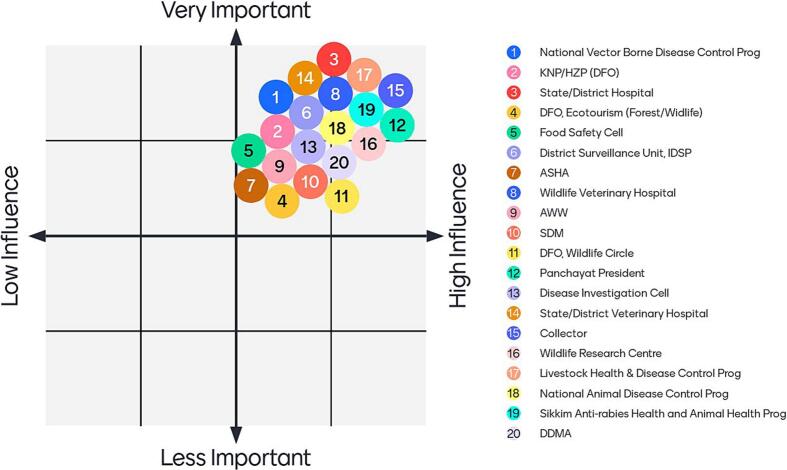
Idealized institutional ordering to tackle zoonoses. 1. National Vector Borne Disease Control Programme; 2. District Forest Officer, Khangchendzonga National Park/Himalayan Zoological Park (KNP/HZP); 3. State/District Hospital; 4. District Forest Officer, Ecotourism (Forest/Wildlife); 5. Food Safety & Standards Cell; 6. District Surveillance Unit, Integrated Disease Surveillance Programme; 7. Accredited Social Health Activist [community health worker]; 8. Wildlife Veterinary Hospital; 9. *Anganwadi* Worker [female nutrition and child health community worker]; 10. Sub-divisional Magistrate; 11. District Forest Officer, Wildlife Circle; 12. *Panchayat* President [local self-government institution]; 13. Disease Investigation Cell; 14. State/District Veterinary Hospital; 15. District Collector/Commissioner; 16. Wildlife Research Centre; 17. Livestock Health & Disease Control Programme; 18. National Animal Disease Control Programme; 19. Sikkim Anti-Rabies and Animal Health Programme; 20. District Disaster Management Authority.

Conceptually there is little to find fault with One Health when it suggests that emerging zoonotic diseases cannot be addressed without addressing disease emergence at the “human-animal-environmental health interface” [41]. The sections above suggest that intersectoral collaboration is not the biggest challenge to addressing zoonoses in a developing country context. In the case of Gyalshing, we observed that inter-sectoral capabilities are already built into the office of the District Collector – the senior-most resident government official in a district – that can be effectively deployed if only existing sectoral institutions are strengthened. There was general consensus on this finding during the workshops conducted during the study (see Fig. 4).

Interviews with officials revealed that in the recent past, Sikkim has witnessed major infrastructure projects, including hydroelectric projects and the construction of airports, highways, and railways that fragment natural habitats. Increased wildlife-human interactions and conflicts were also attributed to the establishment of commercial tea and timber plantations in the past. Fragmented natural landscapes with high human and livestock populations are known to provide the perfect conditions for zoonotic pathogen spill-over [19]. International development projects in the 1970s and 1980s that replaced tropical evergreen forests with cashew plantations, leading to further deforestation for cultivation, housing, and infrastructure, have been linked with emergence of the zoonotic Kyasanur Forest Disease in southern India [19].

In focusing on ‘techno-science solutions’ [14], global One Health guidelines, as they are currently framed, are not only oblivious to institutional capacities in the global South – more dangerously, they keep state efforts focused on reporting outbreaks, rather than navigating attempts to open up these regions for global capital, despite it being a crucial factor for the emergence and re-emergence of zoonoses [18]. In effect, as others have found in similar settings [10], places like Gyalshing, which had little role to play in fomenting the conditions of their susceptibility to zoonoses, are to be treated as little more than warning beacons in service of the global North.

## 7 Conclusion

COVID-19 has significantly increased global attention to zoonoses, in response to which, ‘One Health’ has emerged as an important policy prescription, with numerous endorsements over the past few years. Gyalshing in the state of Sikkim in India, located within a biodiversity hotspot, is predominantly rural and agrarian, with a rising livestock population, and sees large numbers of tourists, while also beset by porous international and state boundaries. These factors engender high incidences of human-animal-forest interactions and the potential for zoonotic disease spillover and transmission. Through our paper we have aimed to convey the dissonances between global One Health mandates and local realities in tackling zoonotic diseases.

The focus of One Health policies is largely on intersectoral coordination and disease reporting [1], which are certainly vital, and in need of strengthening. However, the critical gap in tackling the emergence of zoonoses is the infrastructural and capacity constraints that the sectoral agencies of public health, animal husbandry, and wildlife conservation currently face. This is particularly the case in a developing country setting. A zoonotic disease control programme like SARAH, led by the animal husbandry department, has been successful in controlling both human and canine rabies in Sikkim less because of intersectoral cooperation, but because it produced boots on the ground [35].

Several studies have recognized that prevention, early detection, and containment of emerging diseases in “source” countries requires massive investments in public health care infrastructure [15,42]. Given the scarce resources and challenges facing public and veterinary health in many developing countries, it is unrealistic to expect an integrated One Health system to be overlaid on already burdened governance mechanisms [15,43]. One Health proponents and guidelines cannot afford to be oblivious to these realities. The stakes for incorporating more nuanced formulations of One Health suited to developing country settings have never been higher, as the unfading spectre of COVID-19 continues to remind us. The future of the planet rests on everyone getting this right.

## Author contribution statement

Conception or design of the work – Mridula Mary Paul, Abi T Vanak.

Data collection – Aarti Chettri, Sunita Pradhan, Sarala Khaling, Mridula Mary Paul.

Data analysis and interpretation – Mridula Mary Paul, Sunita Pradhan.

Drafting the article – Mridula Mary Paul.

Critical revision of the article – Sunita Pradhan, Aarti Chettri, Sarala Khaling, Abi T Vanak.

All authors approve of the final version of the paper and submission.

## Reflexivity statement

This paper was written by a multi-disciplinary group of researchers who work in India, about the application of global health policy in an Indian context, with lessons for other developing country settings. Three of the authors are from the state of Sikkim, where the study is located, while the other two are based at the headquarters of the India-based research organization they belong to. One of them has since moved to a University in the UK and another to a University in Sikkim to pursue their doctoral studies. We have a favourable gender ratio, with all the authors except one identifying as female. Across disciplines, there are very few studies that are located in the Indian Himalayan Region, and even fewer that are undertaken by local researchers. This is especially the case in the fields of public health, zoonoses, and One Health. We are happy to therefore present this unique perspective and findings on an issue of global public health relevance.

## 2–4 Key messages, detailing the main points made in the paper

Locally relevant formulations of One Health are needed that suit the context and constraints of developing countries.

Critical reflection on global health policies, and the agenda and politics behind it and who it serves.

Structural violence of international policies, including global health policies, when it emanates from the global North with the aim to order the global South for its own benefit.

## Ethical approval

Ethical approval for this type of study is not required by our institute.

## Author statement

This is to confirm that generative AI and AI-assisted technologies were not used in the writing of the paper “Putting One Health to the Test: Operational challenges and critical reflections from the global South”.

## CRediT authorship contribution statement

**Mridula Mary Paul:** Writing – original draft, Visualization, Validation, Methodology, Investigation, Formal analysis, Data curation, Conceptualization. **Mridula Mary Paul:** Writing – original draft, Visualization, Validation, Methodology, Investigation, Formal analysis, Data curation, Conceptualization. **Sunita Pradhan:** Writing – review & editing, Validation, Supervision, Investigation, Formal analysis, Data curation. **Aarti Chettri:** Investigation. **Sarala Khaling:** Writing – review & editing, Supervision, Project administration. **Abi T. Vanak:** Writing – review & editing, Supervision, Funding acquisition, Conceptualization.

## Declaration of competing interest

The authors confirm that there are no known conflicts of interest associated with this publication and there has been no significant financial support for this work that could have influenced its outcome.

## Data Availability

No data was used for the research described in the article.

## Appendix A Supplementary data

Supplementary material: Questions used for semi-structured interviews & Disease prioritization criteria and scoring Image 1
