# Supplementary material for: Putting one health to the test: Operational challenges and critical reflections from the global South
Source: One Health. 2024 Dec 29;20:100963. doi: 10.1016/j.onehlt.2024.100963 (PMC11773206; doi:10.1016/j.onehlt.2024.100963)
Supplement: Supplementary file 1 — Supplementary material: Questions used for semi-structured interviews & Disease prioritization criteria and scoring [file mmc1.docx]

**Putting One Health to the test: Operational Challenges and Critical Reflections from the Global South**

**APPENDIX I**

**Outline for semi-structured interviews with key informants**

1. The Disease
   1. What are the common zoonotic diseases that occur in your area? (list prepared).
   2. What is the mortality rate due to the disease? (question repeats for each disease mentioned by key informant).
   3. Are there particular areas from where large number of this disease are reported. Is it urban or rural?
   4. Which age groups/gender are most vulnerable?
   5. Which local communities/ group are most vulnerable?
   6. Is this disease seasonal or not, if yes in which season does it occur?
   7. Is treatment available for all? (treatment in government hospitals require Sikkim domicile, hence there are chances that others might be unable to unable to access treatment)
2. Current facilities and infrastructure available for the treatment and management of the disease
3. Challenges in diagnosis, treatment, and management of the disease
4. Suggestions and recommendations

**APPENDIX II**

**Prioritization of Zoonotic Diseases in Sikkim**

| **Criteria & Question** | **Answer** | **Score** |
| --- | --- | --- |
| Criterion A: Intervention Ability  Question: Is there a vaccine or treatment available for humans or animals? | None | 0 |
|  | Only humans | 1 |
|  | Only animals | 2 |
|  | Both humans and animals | 3 |
| Criteria B: Severity of Disease  Question: What is the human Case Fatality Rate of the disease? | <5% | 0 |
|  | 5 - <10% | 1 |
|  | 10 - <50% | 2 |
|  | 50 – 100% | 3 |
| Criteria C: Economic Burden  Question: What is the economic loss in livestock? The disease agent has: | <5% CFR in animals, <20% production loss | 0 |
|  | <5% CFR in animals, ≥20% production loss | 1 |
|  | >5% CFR in animals, <20% production loss | 2 |
|  | >5% CFR in animals, ≥20% production loss | 3 |
| Criteria D: Response Capacity  Is there an established surveillance system | None for animals, none for humans | 0 |
|  | Yes for animals, none for humans | 1 |
|  | None for animals, yes for humans | 2 |
|  | Yes for animals, yes for humans | 3 |
| Criteria E: Transmissibility  Question: What is the human-to-human disease transmission potentiality? The disease agent has: | No potential for human-to-human transmission | 0 |
|  | Rare human-to-human transmission | 1 |
|  | Sustained human-to-human transmission | 2 |
